# Could Mussel Populations Be Differentially Threatened by the Presence of Microplastics and Related Chemicals?

**DOI:** 10.3390/toxics13030181

**Published:** 2025-02-28

**Authors:** Filipe Borges, Rosa Freitas, Ana L. Patrício Silva, Dulce Lucy Soliz Rojas, Gema Paniagua González, Montserrat Solé

**Affiliations:** 1 Departamento de Biologia, Universidade de Aveiro, 3810-193 Aveiro, Portugal; filipeborges@ua.pt (F.B.); rosafreitas@ua.pt (R.F.); ana.luisa.silva@ua.pt (A.L.P.S.); 2 Centro de Estudos do Ambiente e do Mar (CESAM), Universidade de Aveiro, 3810-193 Aveiro, Portugal; 3 Departamento de Ciencias Analíticas, Facultad de Ciencias, Universidad Nacional de Educación a Distancia, 28232 Madrid, Spain; dsoliz@ccia.uned.es (D.L.S.R.); gpaniagua@ccia.uned.es (G.P.G.); 4 Institut de Ciències del Mar-Consejo Superior de Investigaciones Científicas, ICM-CSIC, 08003 Barcelona, Spain

**Keywords:** polyamide, tricresyl phosphate, acetylcholinesterase, antioxidant enzymes, carboxylesterase, plastic additives

## Abstract

Mussels serve as indicators of anthropogenic chemical pollution; however, the effects of microplastics and plastic-related chemicals on their health performance remain an emerging issue. In this study, mussels were exposed to a polyamide (PA; 5 μg/L) and tricresyl phosphate (TCP; 1 μg/L) for 28 days. The exposures to the two contaminants were performed independently or in combination and lasted 28 days. The results showed that the independent exposure altered enzyme activities more significantly than the combined one. Exposure to the PA significantly (*p* < 0.05) inhibited the antioxidant enzyme catalase (CAT) by 43.5% and the neurotransmitter enzyme acetylcholinesterase (AChE) by 40.6%, while TCP specifically inhibited carboxylesterase (CE) activity by 38.5%, all in respect to the solvent control. When both pollutants were combined, most biomarker responses were similar to control levels. To further investigate if the mussels’ response to contaminants (here, chemical compounds only) could be population-specific, a comparative study between Atlantic and Mediterranean mussels was included. Firstly, baseline detoxification defenses were contrasted in the digestive glands of each mussel population, followed by an assessment of in vitro responses to a wide range of plastic additives. The results revealed that Mediterranean mussels expressed higher baseline activities for most detoxification enzymes, although the in vitro sensitivity to the targeted chemicals was similar in both populations. Of all the plastic additives tested, TCP significantly inhibited CE activity both in vivo and in vitro. The in vitro screening also indicated that other plastic additives could act as strong inhibitors of CE. However, additional in vivo exposures in mussels are needed to confirm CE suitability as a biomarker of these chemical exposures. All together, these results also suggest critical population-level differences in susceptibility to microplastic pollution, highlighting a need for targeted conservation efforts.

## 1. Introduction

Environmental pollution is a major consequence of human activities, and marine ecosystems are impacted by physical and chemical entities, including the overwhelming presence of plastics and their derivatives [1]. Because plastic is the most prevalent type of marine litter and may remain in the oceans for a long time, plastic pollution can represent a serious environmental hazard to marine life. While around 80% of total marine plastic originates from inland, either from laundering clothes, littering, solid waste disposal, or plastic bags [2,3], the remaining 20% results from commercial fishing equipment, like fishing nets and lines. Another significant source of plastic debris, particularly microplastics (plastic particles smaller than 5 mm), is aquaculture. This is due to the extensive use of plastic materials in essential infrastructures in these productive activities [4]. Among the most abundant plastic polymers, high-density polyethylene (HDPE), low-density polyethylene (LDPE), polyvinyl chloride (PVC), polyethylene terephthalate (PET), and polyamides (PAs) make up about 90% of all globally produced plastic [5].

Polyamides (PAs) are widely used in industries such as the automotive, fishing, aquaculture, and textile industries [4]. With a density varying from 1.13 to 1.35 g/cm^3^, microplastics of PAs are prevalent in the water column and the seafloor [6]. Microfibers, as a significant industrial application of polyamides (mainly nylon), are abundant in the marine environment, comprising over 90% of microplastics in the Atlantic and 75% in the Pacific Ocean [7,8]. When ingested by marine organisms, such particles can disrupt growth, feeding, reproduction, and survival [9,10,11,12]. The toxicity of microplastics is often due to the particles’ physical characteristics (size, shape, among others) and/or their associated chemical additives, which are incorporated during the plastics’ manufacture to provide desired properties for future applications. For instance, flame retardants are incorporated to increase plastics’ resistance to inflammability [13,14].

Flame retardants (FRs), in particular, are emerging chemicals commonly used in almost all everyday objects, from clothes to home furniture and appliances [15]. From a chemical perspective, they can be divided into halogenated or organophosphorus flame retardants (OPFRs). Due to the negative impacts caused by the halogenated forms on terrestrial and aquatic life [13,16,17,18,19], OPFRs are proposed as presumably less toxic alternatives [20]. Nonetheless, some aryl-OPFRs are reported to interfere with mammalian detoxification processes [21].

Marine sentinel filter feeders, such as bivalves, actively ingest contaminants, including microplastics and plastic additives, which may cause metabolic distress, such as oxidative stress, cellular damage, and neurotoxicity [22,23,24]. For this reason, the U.S. National Oceanic and Atmospheric Administration (NOAA) has a program called “Mussel Watch”, which consists of monitoring the coastal waters using bivalves (mostly mussels and oysters) with the purpose of assessing contaminants [25,26]. Additionally, mussels have a relevant ecological role in the marine food web, as prey for many species, and a commercial interest in aquaculture. Mussels play a key role in marine ecosystems, particularly in coastal areas. As filter feeders, they improve water quality by feeding on suspended particles, such as phytoplankton and organic debris, helping maintain the ecological balance in their environment. However, their vulnerability to microplastic pollution is a growing concern. They can ingest microplastics while filtering the water, which can lead to negative effects, such as the obstruction of their digestive systems, the disruption of their reproductive mechanisms, and even the accumulation of toxic substances in their tissues. Furthermore, these microplastics that affect mussels can enter the food chain, impacting the predators that consume them, including humans [27].

The mussel *M. galloprovincialis* is widely adopted as a bioindicator in toxicological, monitoring, and environmental studies [28]. Moreover, its tough shell and easy handling in the laboratory facilitates experimentation [29]. So far, studies regarding the ingestion of microplastics by mussels indicate that this can lead to histological, inflammatory, and immunological alterations, lysosomal membrane destabilization, a reduced filtering rate, neurotoxicity, oxidative stress, hemocyte mortality, dysplasia, genotoxicity, and transcriptional responses [30,31,32]. Nonetheless, toxicity due to fibers is largely unknown, despite being one of the most frequent forms (>60%) of bioaccumulated microplastics in bivalves [33], including those from PA [34,35]. Furthermore, as aquaculture species, mussels can threaten humans’ health because most cultivated mussels have been shown to have their guts contaminated by microplastics [29,36,37,38].

In toxicology assessment, biochemical biomarkers are adopted as early warning tools to predict harmful consequences at higher physiological and ecological levels, including in bivalves as sentinels [39]. Among the biomarkers adopted in mussel studies, the activities of those involved in phase I of detoxification processes stand out, such as carboxylesterases, phase II glutathione S-transferases involved in conjugation, and the antioxidant defenses: glutathione peroxidase, catalase, and glutathione reductase. Lipid peroxidation has commonly been used as a biomarker to infer oxidative damage and acetylcholinesterase inhibition as an indicator of neurotoxicity [35,40].

Thus, the aim of this study was to assess the effects of polyamide (PA) and tricresyl phosphate (TCP), alone or in combination, on mussels at environmentally relevant concentrations. The targeted toxicological endpoints included alterations in metabolic processes, oxidative stress, and neurotoxicity. Additionally, we explored the in vitro sensitivity of phase I carboxylesterases to plastic additives of environmental concern in two mussel populations (Atlantic and Mediterranean) in order to assess any singular population-specific biochemical adaptability. These results are discussed regarding their respective biochemical activities and the bioaccumulation of plastic additives from the bisphenol and phthalate families.

## 2. Materials and Methods

### 2.1. Mussels’ In Vivo Exposure to Polyamide (PA) and Tricresyl Phosphate (TCP)

Mussels, *Mytilus galloprovincialis*, were collected at the Mira Channel from the Ria de Aveiro in the northwest (NW) of Portugal, which is considered the least impacted channel of this coastal lagoon [41]. After collection, about 75 mussels were transported to the laboratory and placed into 150 L tanks containing artificial seawater (Tropic Marin^®^SEA SALT, Wartenberg, Germany). Their shells were cleaned from algae and barnacles attached to them to ensure water quality. Depuration lasted 3 days, during which food was not given. Yet, throughout the 10 days of acclimation, feed was provided. During the procedure, mussels were kept at 17 ± 1.0 °C, pH 8.0 ± 0.1, and a salinity of 30 ± 1.0, with continuous aeration and a natural photoperiod (16:8 h light–dark), and fed with Algamac protein plus (750 µL/aquarium) every other day. Afterwards, for the exposure, mussels were distributed into different aquaria (5 mussels per aquarium of 3 L) corresponding to the five experimental conditions in triplicate. 

The polyamide microplastics (PA, size from 64 to 125 µm gently provided by a local company that prefers to remain anonymous) and the additive tricresyl phosphate (TCP; CAS 1330-78-5) were added to the respective treatment at the environmentally relevant concentrations: 5 µg PA/L and/or 1 µg TCP/L. Since TCP is not soluble in water, ethanol was used as a solvent and the same concentration of solvent (<0.001%) was used in the carrier control tank (used as a control for further statistical comparison). The four contrasted treatments included the following: (1) solvent control, (2) PA, (3) TCP, and (4) PA and TCP. The experiment was conducted at 17 °C and regularly checked for optimal physicochemical conditions during the 28 days of exposure. Water changes and compound renewal were conducted weekly. At the end of the exposure, of the 5 mussels in each aquarium, 3 were used for biochemical analysis and the other 2 to quantify PA particles in whole tissue.

### 2.2. Sample Preparation and Biochemical Determinations

At the end of the 28-day exposure period, 3 of the 5 (un)exposed mussels in each replicate aquarium (*n* = 9) were dissected and frozen with liquid nitrogen and kept at −80 °C until the homogenization step under liquid nitrogen using a pestle and mortar. About 2.5 g of tissue fresh weight (FW) was divided into 5 aliquots (each of 0.5 g) and preserved for further biochemical analysis.

In total, 1 mL of 100 mM phosphate buffer pH 7.4 containing 150 mM KCl, 1 mM dithiothreitol (DTT), and 1 mM ethylenediaminetetraacetic acid (EDTA) was added to each aliquot in a 1:2 (*w*/*v*) ratio for the analysis of most of the selected parameters, except for LPO measurements, which used trichloroacetic acid (TCA) as the extraction buffer. These included the activities of acetylcholinesterase (AChE), carboxylesterases (CEs) with the substrates p-nitrophenyl acetate (pNPA) and p-nitrophenyl butyrate (pNPB), glutathione *S*-transferases (GSTs), glutathione reductase (GR), glutathione peroxide (GPx) with cumene hydroperoxide (CHP) and hydrogen peroxide (H_2_O_2_) as substrates, catalase (CAT), and total protein (PROT) content.

For each 0.5 g sample weight, 1 mL of the appropriate buffer and a metal ball were added to homogenize the tissue using TissueLyser II (Qiagen, Hilden, Germany) for 90 s at a frequency of 20 1/s, followed by centrifugation at 10,000× *g* for 20 min; these steps were conducted at a temperature of 4 °C. The resulting supernatant (S9) was aliquoted and kept in a −80 °C freezer until analysis. A detailed description of all nine biomarkers tested can be found elsewhere [42,43], and the methodology is briefly described in the Supplementary Information File.

### 2.3. Sample Preparation, Biochemical Determinations, and In Vitro Incubations in Atlantic and Mediterranean Mussels

Mussels from Aveiro’s Atlantic coast and the Mediterranean Adriatic Sea were collected from their respective natural environments. Water parameters for the Atlantic mussels at the sampling site were as follows: temperature (T): 16.65 °C; salinity: 34.9; chlorophyll: 0.33 mg/m^3^; and dissolved oxygen (DO): 247.11 mmol/m^3^. For the Mediterranean mussels, the environmental conditions were as follows: T: 11.86 °C; salinity: 36.94; chlorophyll: 0.77 mg/m^3^; and DO: 275.62 mmol/m^3^. The Atlantic mussels were from the same location as those used for the in vivo experiments (*n* = 15), while the Mediterranean ones, of similar size, were provided by an aquaculture farm near Sant’Elpidio and kept in the ICM-CSIC ZAE facilities in Barcelona in 30 L aquaria for 24 h (*n* = 15). In both cases, mussels were lab-depurated for 24 h before dissection.

To contrast mussel populations, only digestive glands were used for biochemical determinations (*n* = 8) and in vitro contrasts (*n* = 4 each made of 3 pooled mussels). Fresh digestive glands from each mussel population were homogenized with 100 mM pH 7.4 buffer phosphate buffer, containing 150 mM KCl, 1 mM EDTA, and 1 mM DTT at a 1:4 (*w*/*v*) ratio, using a Polytron tissue homogenizer. After that, the homogenate was centrifuged at 10,000× *g* for 30 min at 4 °C (S9). Whole tissue from a few individuals (*n* = 3 pooled samples) was used for the chemical determination of the plasticizers (bisphenols and phthalates).

The S9 fraction from pooled mussels (*n* = 4) in each population was used for in vitro incubations with selected plastic additives of environmental concern at a single 100 µM concentration. In parallel, commercial human recombinants, CE1 (E0162) and hCE2 (E0412) from Sigma-Aldrich (St. Louis, MO, USA), were run in the same microplates to validate the methodological procedure. Incubations lasted 15 min at room temperature, and the residual pNPB-CE activity was measured and referred to as the percentage of the carrier control (100%), as described elsewhere [44]. The chemicals tested in vitro (Table 1) were chosen because of their environmental relevance, their known ability to inhibit specific mammalian CEs, their varied solubility and lipophilia (LogKow), and also their application in other mollusk studies [44,45]. The organophosphorus pesticide BNPP was used as a positive control due to its ability to specifically inhibit CE using pNPB as substrate, and the neonicotinoid BW284c51 was used as a positive control to validate AChE inhibition. A 7-point concentration range of both positive controls, BNPP and BW284c51 (10^−4^ to 10^−10^ M), was used to calculate the IC50 value for pNPB-CE and AChE activities, respectively, in both mussel populations.

### 2.4. Quantification of Polyamide (PA) Microplastics in the Media and the Mussels’ Tissue

Water from the medium (50 mL) was collected at the beginning and the end of the test in each treatment, and two (of the initial five) mussels per condition were used for microplastic quantification following Prata et al.’s 2021 protocol [53], with slight modifications. Briefly, the water was directly vacuum-filtered onto microfiber filters (0.7 µm pore, size 47 mm ∅, ref. A0478855, Prat Dumas, Couze-et-Saint-Front, France), rinsed with hot water, and treated with acetone for 3 min (vacuum off). Afterwards, the filter was washed with milli-Q water, stained with Nile red (10 µg/mL ethanol) for 3 min, thoroughly washed with milli-Q water, and stored in ∅ 60 mm Petri dishes until completely dry at room temperature for further microscopic analysis.

The soft tissue of mussels was frozen at −20 °C in glass vials and lyophilized for 72 h. Afterwards, the samples were subjected to chemical digestion with KOH 10% (1:3 *w*/*v*) for 48 h at 50 °C, followed by additional treatment with H_2_O_2_ 10% for 24 h at room temperature (19 °C). After digestion, samples were vacuum-filtered onto the formerly described microfiber filter to retain the microplastics. After filtration, the procedure was the same for water samples. All samples were covered with aluminum foil to avoid airborne contamination, and blanks between samples were included to prevent cross-contamination. The quantification of PA microplastics was conducted under a stereomicroscope (MS5, Leica Microsystems, Houston, TX, USA) coupled with a camera (EOS M50 Cannon, Canon Inc., Uxbridge Middlesex, UK). For detection, filters were enlightened with a blue light (470 nm), and photos were taken under an orange filter; this procedure allowed PA microplastics stained with Nile red to appear as red fluorescent particles.

### 2.5. Plastic Additive Analysis in Atlantic and Mediterranean Mussels

A simple and efficient solid-phase dispersion extraction (MSPD) method was applied in order to determine six plastic additives, bisphenol S (BPS), bisphenol F (BPF), bisphenol A (BPA), di(2-ethylhexyl)phthalate (DEHP), dibutyl phthalate (DBP), and diethyl phthalate (DEP), in the whole tissue of mussels from Atlantic and Mediterranean waters [54]. Homogenized samples (0.1 g), previously spiked with the analytes, were mixed with 0.5 g of Florisil sorbent in a mortar. After 10 min of manual grinding, the homogeneous mixture was transferred into a glass cartridge containing a plug of silanized glass wool at the bottom. The analytes were extracted using 9 mL of MeOH/MeCN (70:30, *v*/*v*) in three static extraction steps. The extract was evaporated at room temperature under a nitrogen stream, and the residue was dissolved in 400 μL of an 85:15 (*v*/*v*) MeOH/H_2_O mixture. A blank sample was prepared following the same procedure but without spiking the MSPD mixture with the analytes. The prepared samples were analyzed by HPLC-ESI-MS, following the methodology described in previous works [55].

### 2.6. Statistical Analysis

No significant differences in biochemical responses were observed between the mussels in the control and solvent-control groups (Student’s *t*-test), except for lipid peroxidation (LPO). Therefore, differences between the following treatments were analyzed using a one-way ANOVA, followed by Tukey’s post hoc multiple comparison tests: (1) solvent control, (2) PA, (3) TCP, and (4) PA + TCP; *n* = 9 in each condition.

No statistics were conducted for the in vitro tests with commercial purified proteins, as they corresponded to a single biological sample. In the case of the Atlantic and Mediterranean contrasts, four biological pooled samples for each population were considered, and the statistical significance was considered when the inhibition was greater than 20% [48]. Baseline activity contrasts of the two mussel populations were performed using Student’s t-test (*n* = 8). Statistical significance was set at *p* < 0.05. All analyses were conducted using GraphPad Prism v.6.

## 3. Results and Discussion

### 3.1. Media Characteristics and PA Microplastic Ingestion by Atlantic Mussels

The water quality parameters (pH, T, salinity, DO) remained stable during exposure, namely, pH: 8.0 ± 0.1; T 17 °C ± 1.0; salinity 30 ± 1.0; and DO > 90%. The concentration of PA microplastics in the water column also remained stable (day 0: 748 ± 40 items/L; day 28: 840 ± 66 items/L). The mussels presented internal concentrations of 4 ± 2 items/organism in the case of the solvent control, 17 ± 9 items/organism in the case of the PA-exposed mussels, and 13 ± 3 items/organism under the combined exposure, which confirmed the occurrence of PA microplastics in the 28-day-exposed mussels. In field mussels from the Portuguese coast, fibers constituted 39% of the identified microplastics, and among them, PA represented 12% of this polymer nature [34]; however, to the best of our knowledge, no study to date has addressed the exposure of mussels to PA and TCP in realistic environmental concentrations.

### 3.2. Effects of In Vivo Exposure to PA Microplastics and TCP in Mussels

**Figure 1:**
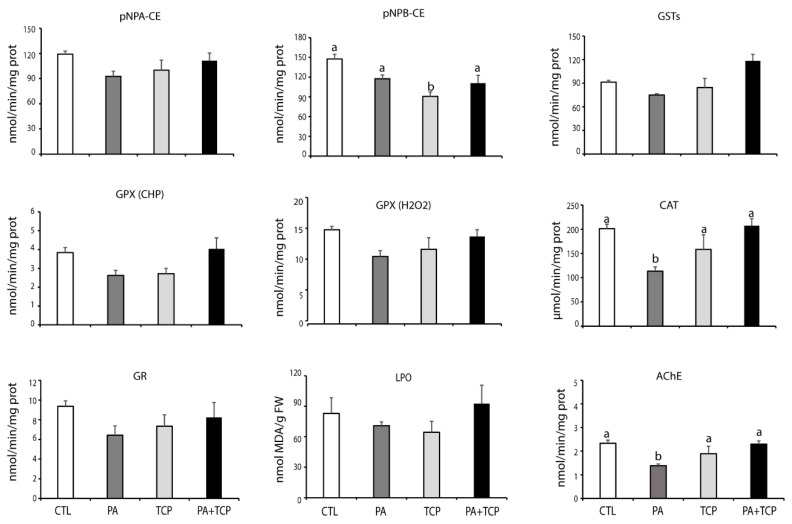
Enzyme activities (mean ± SEM; *n* = 9) in the whole tissue of Atlantic mussels exposed to polyamide (PA) microplastics, tricresyl phosphate (TCP), and both under a lab exposure that lasted 28 days. Acronyms as in the Materials and Methods Section. Different letters denote a statistical difference versus the solvent control (CTL).

The targeted biomarkers were measured in the whole tissue of mussels exposed for 28 days to the solvent control, PA microplastics (5 mg/L), TCP (1 µg/L), and PA microplastics and TCP in combination. The results on these biochemical parameters are depicted in Figure 1, with statistical support shown in Tables S1 and S2 (Supplementary Information).

Exposure to the PA microplastics caused a significant (*p* < 0.05) inhibition in CAT (43.5%) and AChE (40.6%) activities, while exposure to TCP caused a significant inhibition of pNPB-CE (38.5%). Other inhibitions caused by PA exposure in respect to the solvent-control mussels varied from 17.9 (GST) to 31% (GR and GPX), but due to a large variability in the responses, they did not reach significance (*p* > 0.05). Likewise, TCP inhibited most enzymatic parameters at a mean of 20% without reaching statistical significance either. A general trend was observed where most enzyme activities were lower under independent PA and TCP exposures, with activities similar to control values under co-exposure (Figure 1). It can be postulated that the combined exposure set up the mechanistic defenses to face these more stressful conditions and/or that the action of both chemicals was antagonistic, with both mechanisms not excluding each other. Under co-exposure, unpredicted results can be obtained: additive, antagonistic, and synergistic effects either enhancing the effects or masking them. In the present study, an unexpected outcome took place, as co-exposure usually predicts increased toxicity. This was the case with mammalians exposed to MP, including PA, associated with metals such as Cd [56] or in fish models exposed to Cd [57]. In the mussel species *Mytilus coruscus*, co-exposure to a microplastic/nanoplastic mixture together with a polyaromatic hydrocarbon (phenanthrene at several doses) was more significant in gill and hemocyte markers than when the exposures were conducted independently [58]. In *M. galloprovincialis*, co-exposure to microplastics of several natures and Cd affected antioxidant defenses and digestive enzymes in respect to controls, but co-exposure did not result in a greater harm than independent microplastics and Cd alone, more in line with our observations [37]. The ingestion of PA microfibers alone affected biochemical endpoints: the antioxidant SOD increased in mussels’ (*Mytilus* spp.) digestive glands after 24 h exposure but returned to control levels after 7 days [59]. Other studies where the invertebrate *Ciona robusta* was exposed to NPs and the plastic additive BPA revealed that co-exposure did not enhance the BPA toxicity seen as AChE inhibition [60], presumably revealing an antagonistic effect, more in line with our mussel observations. Another study where zebrafish were exposed to polystyrene nanoplastics (PS-NPs) and an antioxidant plastic additive revealed that co-exposure was more harmful to males than females [61], thus being sex-dependent. All these studies evidence the complexity of the outcomes under co-exposure and the fact that an anticipated enhanced toxicity under co-exposure is not always confirmed. However, it must be taken into consideration that we limited our study to a few endpoints mostly related to detoxification processes, and other physiologically relevant ones that we did not target could have been impaired.

Exposure to the same brand and size of PA but at a higher concentration (1 mg/L) targeted common parameters (CAT, GSTs) in gills and digestive glands in the same mussel species, under the co-occurrence of 2% of exuded compounds from an invasive seaweed [62]. The results revealed enhanced damage to proteins as carbonyl formation after 3-day co-exposure, despite CAT and GSTs not being altered in this shorter time frame. This emphasizes the complexity of the responses under co-exposure when chemicals, doses, targeted tissues, and exposure time differ, even if the endpoints and the sentinel species are coincident.

To the best of our knowledge, no exposure to any aryl-OPFRs has been conducted in mussels. However, the halogenated OPFR, tris(1,3-dichloro-2-propyl) phosphate (TDCPP), also of environmental concern, has been targeted at a relevant dose (10 µg/L) and time frame (28 days) [63]. In this reported case, AChE activity in gills was inhibited after 7 and 28 days of exposure, while GSTs were not affected. Proteomic analysis in gills revealed that the detoxification processes were significantly affected, although CEs were not targeted [63]. In another study with embryos of zebrafish, *Danio rerio*, in vivo TCP exposure alone inhibited the gene expression of *ces2* in 5 dpf exposed fish, and this was also the case for its corresponding in vitro CE activity using whole-tissue homogenates of unexposed fish (Sole et al., personal observation). Indeed, *ces2* gene expression in the TCP-exposed larvae was downregulated in a dose-dependent manner with a significant 2.8-fold change at the highest TCP concentration, together with the downregulation of other genes involved in the immune response. All these former studies and our observation in mussels confirm that TCP, and likely other aryl-OPFRs, interfere with the detoxification processes of invertebrates in the same way as has been seen in vertebrates. Likewise, in vitro, exposure to TCP and other aryl-OPFRs was revealed to interfere with hepatic CE not only in zebrafish larvae but also in other vertebrate and many invertebrate species, including recombinant human CEs [45,64] and microsomal hepatic human CEs [21]. More in vivo exposures at realistic concentrations and in a broader range of marine organisms are needed to validate the suitability of CE as biomarker of OPFR exposure.

### 3.3. Enzyme Activities in Atlantic and Mediterranean Mussels and Their In Vitro Sensitivity to Plastic Additives

**Figure 2:**
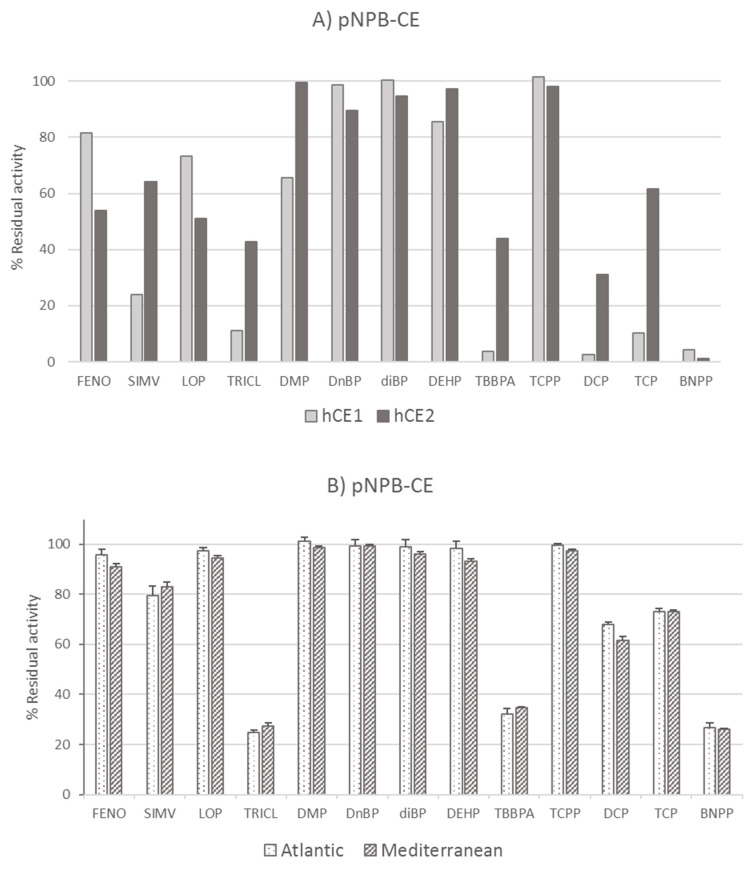
In vitro inhibitory action of several drugs and plastic additives on carboxylesterase hydrolysis rates using p-nitrophenyl butyrate (pNPB) as substrate concerning carrier control (100%). (**A**) Using recombinant human CE1 and CE2 and (**B**) digestive gland S10 homogenates from Atlantic and Mediterranean mussels (*n* = 4). Full names and details of the chemicals are shown in Table 1. All exposures lasted 15 min and correspond to a 100 µM concentration of the targeted chemicals.

Susceptibility to environmentally relevant chemicals in two mussel populations, Atlantic and Mediterranean, was assessed in their digestive gland S9 homogenates by means of (1) the determination of basal enzyme activities involved in detoxification, oxidative stress, and neurotoxicity and (2) in vitro inhibition by a wide range of chemicals of environmental concern (Table 1) at a single 100 µM concentration. This comparative in vitro screening also included commercially purified proteins and drugs of known inhibitory potential to validate the methodological conditions (Figure 2). In other invertebrates (i.e., terrestrial insects and crayfish), a population-specific tolerance to chemicals has been reported [65], but to the best of our knowledge, a particular biochemical adaptability has not been described in mussel populations.

The basal activities of detoxification biomarkers in the digestive glands of both mussel populations identified that phase I and II enzymes and antioxidant defenses (except total GPX) were higher in Mediterranean mussels (Table 2). The ratio of AChE/CE was also lower in mussels from the Mediterranean population, which is regarded as a sign of higher protection in a given tissue when facing neurotoxic chemicals [66]. To back up the presumption that these activity differences were strictly population-specific and not due to a different chemical exposure, a selection of plastic additives from the bisphenol and phthalate families was determined in these mussels’ whole-body tissue as a proxy for chemical exposure. Nevertheless, other non-targeted chemicals could be differentially accumulated in the two mussel populations.

The bioaccumulation of plastic additives in Atlantic and Mediterranean mussels revealed the presence of the bisphenol analogues BPS and BPF, while BPA was <LOQ (Table 3). The phthalates identified were DEH, DBP, and DEHP. The bioaccumulation trend in Atlantic mussels was DEHP > DEP > DBP > BPS > BPF, with concentrations ranging from 0.271 ± 0.111 to 2.861 ± 0.362 µg/g d.w, whereas, in Mediterranean mussels, there was a slightly different trend, BPS > DEHP > BPF > DBP > DEP, but a similar range from 0.109 ± 0.001 to 2.439 ± 0.084 µg/g d.w. These concentrations are in line with those reported for the same mussel species near an aquaculture facility on the Mediterranean island of Mallorca (from 0.146 ± 0.009 to 6.395 ± 2.681 µg/g d.w.) [55] using the same analytical methodology. Comparative results with other mussels (Table 3) indicate that the highest concentrations of plastic additives such as BPA (6.395 ± 2.681 µg/g d.w.) and DBP (1.804 ± 0.203 µg/g d.w.) in samples from Mallorca may reflect the wide use of plastic material in these activities, while BPA was not detected in the mussels in our study. Further contrasts with mussels from other Spanish geographical areas (Table 3) indicate that bisphenols showed the lowest concentrations of BPS, BFF, and BPA in mussels from the Galician Rias [67] and Bay of Biscay [68] in the Atlantic and Coast of Granada in the Mediterranean [69]. For phthalates, the bioaccumulation results were similar in Atlantic and Mediterranean mussels.

Additionally, the in vitro sensitivity of a key detoxification enzyme, pNPB-CE, to plastic additives of emerging concern was contrasted in the two mussel populations, presumably under similar field chemical loads. For methodological validation, human recombinant CE and the digestive gland S9 fraction of both mussel populations were simultaneously contrasted (Figure 2). The rationale for including recombinant hCE1 and hCE2 in the validation protocol is as mentioned above (Table 1). As expected, stronger inhibitions were seen for BNPP (>95%) but also for the flame retardants TBBPA, DCP, and TCP, mostly on the hCE1 isoform (>90%). Other significant inhibitory actions on basal pNPB-CE activity were due to the antimicrobial agent TRICL (88.9%) and the phthalate DMP (34.3%), all on the hCE1 isoform. Interestingly, out of the two halogenated flame retardants, TBBPA was able to strongly inhibit hCE1 (96.4%), while TCPP did not act on any isoform (Figure 2A). At the same in vitro concentration of 100 µM, the response of mussel S9 digestive gland homogenates was comparable to the response previously described for BNPP, TCS, and TBBPA [44] and presently extended to the OPFRs DCP and TCP. A significant inhibition of about 30% for DCP and TCP was confirmed in the mussels, while in the purified hCE1, it was over 90%. This discrepancy of lower inhibitory potential under complex mixture exposure is a recurrent observation when using complex tissue homogenates [70] rather than using the purified protein [44]. Nonetheless, for all plastic additives and the model pesticide (BNPP), no significant differences in the degree of CE inhibition between Mediterranean and Atlantic mussel populations were observed, as it was ~74% after BNPP and between 65.4 and 67.8% after TCS and TBBPA incubations or about 30% for DCP and TCP. Likewise, the IC50 calculations for the two selected enzymes, pNPB-CE and AChE, exposed in vitro to a 7-point range (10^−10^ to 10^−4^ M) of their respective model inhibitors, BNPP and BW284c51, yielded similar IC50 values. For pNPB-CE, it was 8.8 × 10^−5^ ± 7.8 × 10^−5^ (Atlantic) and 7.0 × 10^−5^ ± 4.9 × 10^−5^ (Mediterranean), while for AChE, the IC50 was 4.0 × 10^−6^ ± 4.5 × 10^−7^ (Atlantic) and 3.2 × 10^−6^ ± 5.1 × 10^−7^ (Mediterranean). A higher AChE affinity for the nicotinoid BW284c51 than that of CE for BNPP and a more rigorous Michaelis–Menten fit for AChE was also formerly indicated in other bivalves [25,71]. Thus, it can be assumed that, from an enzymatic perspective derived from in vitro exposure, both populations are equally sensitive; however, since the detoxification enzymes (CE and GST), and some antioxidant defenses, were higher in the Mediterranean population, and their susceptibility ratio AChE/CE was lower, these mussels could potentially be more protected in the face of further chemical inputs. In any case, this hypothesis would need confirmation under realistic in vivo simultaneous exposure conditions of both mussel populations.

### 3.4. Ecological Implications

It has been reported that the ingestion of microplastics and their associated additives can cause biochemical changes in mussels, such as alterations in their metabolism or their ability to properly filter nutrients and eliminate pollutants, which could have a negative impact on their ecosystem. On the other hand, mussels are an important food source for many species, including fish and seabirds, and a protein source in many cultures. Therefore, their contamination can affect their predators and even human health, leading to an imbalance in the food chain. Furthermore, microplastics serve as vectors for other pollutants, such as heavy metals and toxins, highlighting the importance of monitoring the health of mussel populations [72]. Thus, the biochemical changes in mussels not only affect the mussels themselves but can also have significant repercussions on marine ecosystems and human health. This impact underscores the need to prioritize research on the vulnerability of key sentinel species like *Mytilus galloprovincialis* to microplastic pollution. Understanding how this affects marine organisms and their ecosystem is essential for implementing effective conservation strategies and policies to reduce plastic pollution in oceans.

## 4. Conclusions

The exposure of Atlantic mussels to environmental concentrations of PA microplastics and the flame retardant TCP caused a significant inhibition of the activities of CAT and AChE (PA) and pNPB-CE (TCP). A contrast of the detoxification enzymes in the digestive glands of Atlantic and Mediterranean mussels revealed that the Mediterranean population exhibited a higher detoxification capacity despite a similar bioaccumulation of plastic additives from the bisphenol and phthalate groups, thus suggesting that the biomarker responses were likely population-specific. Nonetheless, since their in vitro sensitivity to chemicals of environmental concern, in terms of AChE and pNPB-CE activities, was similar, we anticipate that the Mediterranean mussel population could be better equipped in the face of further chemical insults; however, this needs further in vivo confirmation. The in vitro results support the potential of CE activities as biomarkers of exposure to chemicals such as TCS, TBBPA, and aryl-OPFRs, although, to confirm population sensitivity differences, further in vivo experiments are needed to validate CEs as biomarkers.

## Figures and Tables

**Table 1:** List of the compounds used for the in vitro inhibition tests and their reported action on mammalian carboxylesterase 1 (CE1) and carboxylesterase 2 (CE2) using in vitro models.

Acronym	Full Name	CAS Number	Log Kow	Molecular Mass	Formula	B-Esterase Inhibitor	Reference
*Diagnostic inhibitors*							
BNPP	Bis(4-nitrophenyl) phosphate	645-15-8	2.51	340.2	C_12_H_9_N_2_O_8_P	CE	[46]
BW284c51	1,5-bis(4-allyldimethylammoniumphenyl) pentan-3-one dibromide	402-40-4	3.91	406.6	C_27_H_38_N_2_O^2+^	AChE	[46]
*Pharmaceutical drugs*							
Feno	Fenofibrate	49562-28-9	5.19	360.8	C_20_H_21_ClO_4_	CE2 > CE1	[47]
SIM	Simvastatin	79902-63-9	4.68	418.6	C_25_H_38_O_5_	CE1 and CE2	[48]
Lope	Loperamide	34552-83-5	5.15	477.1	C_29_H_33_ClN_2_O_2_	CE2	[49]
*Plastic additives*							
TCS	Triclosan	3380-34-5	4.76	289.5	C_12_H_7_Cl_3_O_2_	CE1 and CE2	[50]
DMP	Dimethyl phthalate	131-11-3	1.60	194.2	C_6_H_4_-1,2-(CO_2_CH_3_)_2_	Metabolized by CEs	
DnBP	Dibutyl phthalate	84-74-2	4.50	278.3	C_6_H_4_-1,2-[CO_2_(CH_2_)_3_CH_3_]_2_		[51]
DiBP	Diisobutyl phthalate	84-69-5	4.11	278.3	C_6_H_4_-1,2-[CO_2_CH_2_CH(CH_3_)_2_]_2_		
DEHP	Di(2-etilhexyl)phthlate	117-81-7	5.03	390.6	C_6_H_4_-1,2-[CO_2_CH_2_CH(C_2_H_5_)(CH_2_)_3_CH_3_]_2_		
TBBPA	Tetrabromobisphenol A	79-94-7	4.50	543.9	C_15_H_12_Br_4_O_2_	CE2	[52]
TCPP	Tris(1-chloro-2-propyl) phosphate	13674-84-5	2.59	327.6	-		
DCP	Diphenyl p-tolyl phosphate	26444-49-5	5.25	340.3	C_19_H_17_O_4_P	CE1 and CE2	[21]
TCP	Tricresyl phosphate	1330-78-5	6.34	368.4	C_21_H_21_O_4_P	CE1 and CE2	[21]

**Table 2:** Contrasted enzymatic activities in digestive glands of Atlantic and Mediterranean mussels. Values are mean ± SEM (*n* = 8). Units: nmol/min/mg prot except CAT in µmol/min/mg prot. Student’s t-test *p*-value. Significance set at *p* < 0.05.

Biomarker	Atlantic	Mediterranean	*p*-Value
CAT	24.69 ± 2.96	76.08 ± 7.9	*p* < 0.0001
GPX (CHP)	12.41 ± 0.58	12.42 ± 1.01	*p* = 0.9941
GPX (H_2_O_2_)	6.13 ± 0.33	5.93 ± 0.40	*p* = 0.7105
GR	20.09 ± 2.03	26.35 ± 2.05	*p* = 0.0475
GST	67.68 ± 6.35	123.82 ± 10.38	*p* = 0.0004
pNPA-CE	45.12 ± 3.03	97.57 ± 5.84	*p* < 0.0001
pNPB-CE	99.18 ± 9.75	185.51 ± 13.10	*p* = 0.0001
AChE	1.91 ± 0.16	3.02 ± 0.18	*p* = 0.0003
AChE/pNPA-CE	0.043 ± 0.002	0.032 ± 0.003	*p* = 0.0055
AChE/pNPB-CE	0.020 ± 0.002	0.017 ±0.001	*p* = 0.0784

**Table 3:** Concentration of selected bisphenols and phthalates in the whole tissue of mussel *Mytilus galloprovincialis* from different Spanish Atlantic and Mediterranean areas in this study and in the literature. Present results correspond to mean value ± SD (*n* = 3) in µg/g dry weight or * per wet weight (w.w). NA: not analyzed. <LOQ: lower than the limit of quantification.

Location	Bisphenols			Phthalates			
Atlantic coast	BPS	BPF	BPA	DEP	DBP	DEHP	Reference
	0.854 ± 0.167 *	0.237 ± 0.097 *	<LOQ	1.098 ± 0.057*	0.863 ± 0.089 *	2.499 ± 0.316 *	This study
	0.978 ± 0.191	0.271 ± 0.111		1.257 ± 0.065	0.988 ± 0.102	2.861 ± 0.362	
Mediterranean coast	2.130 ± 0.073 *	1.097 ± 1.084 *	<LOQ	0.095 ± 0.001 *	0.127 ± 0.008 *	1.569 ± 0.200 *	This study
	2.439 ± 0.084	1.256 ± 1.241		0.109 ± 0.001	0.149 ± 0.011	1.797 ± 0.229	
Atlantic coast and Bay of Biscay	NA	NA	0.003–0.714	NA	NA	NA	[68]
Balearic Islands (Mallorca)	0.233 ± 0.441 *	1.942 ± 0.752 *	5.561 ± 2.331 *	0.127 ± 0.008 *	1.569 ± 0.200 *	1.493 ± 1.512 *	[55]
	0.267 ± 0.507	2.233 ± 0.865	6.395 ± 2.681	0.146 ± 0.009	1.804 ± 0.230	1.717 ± 1.739	
Mediterranean coast of Granada	NA	NA	0.011–0.173	NA	NA	NA	[69]
Galician Rias	0.0142–0.138	0.0014–0.139	0.0022–0.185	NA	NA	NA	[67]

## Data Availability

The raw data supporting the conclusions of this article will be made available by the authors on request.

## Supplementary Materials

The following supporting information can be downloaded at: https://www.mdpi.com/article/10.3390/toxics13030181/s1, Table S1: Comparison of biomarker responses in mussels between control and solvent-control groups, using a Student’s *t*-test; Table S2: Statistical results (one-way ANOVA) including the sum of squares (SS), degrees of freedom (df), mean square (MS), F-value, and *p*-value for biochemical analysis in Mytilus galloprovincialis exposed to PA, TCP, and PA with TCP for 28 days. Significant values (*p* < 0.05) are in bold. [73,74,75,76,77,78,79,80]
