# Supplementary material for: Could Mussel Populations Be Differentially Threatened by the Presence of Microplastics and Related Chemicals?
Source: Toxics. 2025 Feb 28;13(3):181. doi: 10.3390/toxics13030181 (PMC11945407; doi:10.3390/toxics13030181)
Supplement: Supplementary file 1 [file toxics-13-00181-s001.zip › toxics-3474914-supplementary.pdf]

toxics

MDPI

Supplementary Information

Could Mussel Populations Be Differentially Threatened by the Presence of Microplastics and Related Chemicals?

Filipe Borges [1], Rosa Freitas [1,2], Ana L. Patrício Silva [1,2], Dulce Lucy Soliz Rojas [3], Gema Paniagua González [3] and Montserrat Solé [4,*]

[1] Departamento de Biologia, Universidade de Aveiro, 3810-193 Aveiro, Portugal; filipeborges@ua.pt (F.B.); rosafreitas@ua.pt (R.F.); ana.luisa.silva@ua.pt (A.L.P.S.)
[2] Centro de Estudos do Ambiente e do Mar (CESAM), Universidade de Aveiro, 3810-193 Aveiro, Portugal
[3] Departamento de Ciencias Analíticas, Facultad de Ciencias, Universidad Nacional de Educación a Distancia, 28040 Madrid, Spain; dsoliz@ccia.uned.es (D.L.S.R.); gpaniagua@ccia.uned.es (G.P.G.)
[4] Institut de Ciències del Mar, ICM-CSIC, 08003 Barcelona, Spain
* Correspondence: msole@icm.csic.es

Academic Editor(s): Antonietta Santoro

Received: 27 January 2025
Revised: 21 February 2025
Accepted: date 25 February 2025
Published: date

Citation: Borges, F.; Freitas, R.; Silva, A.L.P.; Soliz, D.L.; Paniagua González, G. Solé, M. Could Mussel Populations Be Differentially Threatened by The Presence of Microplastics and Related Chemicals? *Toxics* **2025**, *13*, x. https://doi.org/10.3390/xxxxx

Copyright: © 2025 by the authors. Submitted for possible open access publication under the terms and conditions of the Creative Commons Attribution (CC BY) license (https://creativecommons.org/licenses/by/4.0/).

Table S1: Comparison of biomarker responses in mussels between control and solvent-control groups, using a Student's t-test.

Biomarker	t value	df	*p*-value
CAT	1.428	4	0.2265
GPx (H_2O_2)	1.855	4	0.1371
GPx (CHP)	1.600	4	0.1849
GR	0.8538	4	0.4413
GST	1.021	4	0.3651
CbEs (*p*NPA)	1.192	4	0.2992
CbEs (*p*NPB)	2.140	4	0.0990
LPO	3.823	4	**0.0187**
AChE	0.4982	4	0.6445

Significant values ($p < 0.05$) are in bold.

Table S2: Statistical results (one-way ANOVA) including the sum of squares (SS), degrees of freedom (df), mean square (MS), F-value, and *p*-value for biochemical analysis in *Mytilus galloprovincialis* exposed to PA, TCP, and PA with TCP for 28 days. Significant values ($p < 0.05$) are in bold.

**CAT**	*SS*	*df*	*MS*	*F* (DFn, DFd)	*p*-value
Treatments	19262	3	6421	F (3,8) = 7.584	**0.0100**
Residual	6673	8	846.6	-	-
Total	26035	11	-	-	-
**GPx (H_2O_2)**	*SS*	*df*	*MS*	*F* (DFn, DFd)	*p*-value
Treatments	34.46	3	11.49	F (3,8) = 2.662	0.1193
Residual	34.52	8	4.315	-	-
Total	68.97	11	-	-	-
**GPx (CHP)**	*SS*	*df*	*MS*	*F* (DFn, DFd)	*p*-value
Treatments	4.881	3	1.627	F (3,8) = 3.791	0.0585
Residual	3.434	8	0.4292	-	-
Total	8.315	11	-	-	-
**GR**	*SS*	*df*	*MS*	*F* (DFn, DFd)	*p*-value
Treatments	14.10	3	4.698	F (3,8) = 1.289	0.3426
Residual	29.15	8	3.644	-	-

Total	43.25	11	-	-	-
**GSTs**	*SS*	*df*	*MS*	*F* (DFn, DFd)	*p*-value
Treatments	3104	3	1035	*F* (3,8) = 6.500	**0.0154**
Residual	1273	8	159.2	-	-
Total	4377	11	-	-	-
**CbEs (*p*NPA)**	*SS*	*df*	*MS*	*F* (DFn, DFd)	*p*-value
Treatments	1245	3	415.1	*F* (3,8) = 2.001	0.1926
Residual	1660	8	207.5	-	-
Total	2905	11	-	-	-
**CbEs (*p*NPB)**	*SS*	*df*	*MS*	*F* (DFn, DFd)	*p*-value
Treatments	5004	3	1668	*F* (3,8) = 8.392	**0.0075**
Residual	1590	8	198.7	-	-
Total	6594	11	-	-	-
**LPO**	*SS*	*df*	*MS*	*F* (DFn, DFd)	*p*-value
Treatments	1749	3	583.0	*F* (3,8) = 1.235	0.3591
Residual	3777	8	472.1	-	-
Total	5526	11	-	-	-
**AChE**	*SS*	*df*	*MS*	*F* (DFn, DFd)	*p*-value
Treatments	1.760	3	0.5868	*F* (3,8) = 5.623	**0.0227**
Residual	0.8347	8	0.1043	-	-
Total	2.595	11	-	-	-

Enzymatic determinations

All enzymes were analyzed in post-mitochondrial supernatant (PMS) or S9 fraction and expressed as total protein content, which was determined using bovine serum globulin as a standard according to the Bradford method (Bradford, 1976) [74]. Lipid peroxidation was analyzed in the initial homogenate and expressed as organism fresh weight. The specific procedure details are as follows:

Catalase (Cat): CAT activity was determined using the Aebi (1974) [73] method. Initially, 10 µL of the undiluted sample or 10 µL of sample buffer for the blank was placed in triplicate into the microplate. The reaction mixture (RM) consisted of the sample and

30 % (w/v) hydrogen peroxide (H_2O_2) in phosphate buffer 100 mM pH 6.5 (50 mM H_2O_2 in well). The Grenier UV microplate was used, and absorbance was read at 240 nm after 1 min in a TECAN Infinite 200. The results are expressed in µmol *per* min *per* mg of protein.

Glutathione reductase (GR): Based on Carlberg and Mannervik's (1985) [76] method. To this end, 20 µL of sample or buffer (blank) was mixed with 200 µL RM that contained glutathione disulfide (GSSG) and NADPH each at 1 mM in a well with phosphate buffer 100 mM pH 7.4. Absorbance was read at 340 nm after 3 min in the TECAN reader. The Lambert–Beer Law method was used to calculate the final concentration using an extinction coefficient of 6.22 x 10^3 M^{-1} cm^{-1}, expressed in nmol *per* min *per* mg of PROT.

Glutathione peroxidase (GPx): The assay was carried out using the Gunzler and Flohé (1985) [80] method. In each well, 10 µL of sample or buffer as blank was mixed with the reaction mixture for the two substrates: 5 mM cumene hydroperoxide (CHP) for total GPX activity and 0.019 % hydrogen peroxide (H2O2) for the Se-dependent form. To measure the GPx (CHP), the RM contained NADPH, GSH, and GR all in phosphate buffer 100 mM pH 7.4. The final well concentrations were 0.292 mM (NADPH), 2.5 mM (GSH), and 1 U/mL (GR), incubation lasted 2 min, and afterwards, 30 µL of CHP substrate or H_2O_2 was added (0.625 mM in well). To measure the Se-GPX form, the reaction mixture contained 0.420 mM (NADPH), 2.5 mM (GSH), 1.038 mM sodium azide (NaN_3), and 1 U/mL (GR) in phosphate buffer 100 mM pH 7.4. After 2 min incubation, 30 µL of H_2O_2 was added (2.81 mM in well). Following the Lambert–Beer Law, with an extinction coefficient of 6.22 x 10^3 M^{-1} cm^{-1}, the results are expressed in nmol/min/mg of PROT, and absorbance was read at 340 nm after 3 min.

Glutathione S-transferases (GSTs): The activity of GSTs was determined following Habig et al. (1974) [78]. In triplicate, 25 µL of samples (conveniently undiluted), as well as the blank (sample buffer), was mixed with 1 mM 1-chloro-2,4- dinitrobenzene (CDNB) and 1 mM reduced glutathione (GSH) in a well with phosphate buffer 100 mM pH 7.4. The absorbance was read with a TECAN reader at 340 nm after 5 min. The Lambert–Beer Law method was used for the calculation, using an extinction coefficient 9.6 x 103 $M^{-1}cm^{-1}$, and the results are expressed in nmol/min/mg of PROT. The values were obtained because of the reaction between the CDNB and GSH that formed the thioether.

Carboxylesterases (CbEs): For CbEs, the method used came from Hosokawa and Satoh (2001) [79], using 100 mM p-nitrophenyl acetate (pNPA) and 100 mM p-nitrophenyl butyrate (pNPB) as stock substrate solutions. In total, 25 µL of sample or buffer for blank was mixed with 200 µL of RM that contained the substrates in a well with final concentrations of 1 mM pNPA or pNPB in phosphate buffer 50 mM pH 7.4. Absorbance was read at 405 nm after 5 min and is expressed in nmol *per* min *per* mg of protein.

Acetylcholinesterase (AChE): AChE activity was assayed using acetylcholine (ATC) as substrate using the Ellman et al. (1961) [77] method. In triplicate, 25 µL of sample or buffer was mixed with 150 µL of DTNB (5,5′-Dithiobis-(2-nitrobenzoic acid)), and after 2 min incubation, 50 µL of ATC was added. The well concentrations were as follows: 0.18 mM (DTND) and 1mM (ATC). Absorbance was read at 412 nm after 5 min. Activity is expressed in nmol *per* min *per* g of protein, using a molar extinction coefficient of 13.600 M^{-1} cm^{-1}.

Lipid peroxidation: Followed the Buege et al. (1978) [75] method using trichloroacetic acid (TCA) 20 % (w/v) and 2-thiobarbituric acid (TBA) 0.5 % (w/v) as substrate. The levels were assessed by quantifying the concentration of malondialdehyde (MDA). Firstly, 100 µL of sample, which was already extracted by TCA as extracting buffer, was placed in a microtube or blank. Then, 400 µL of TBA and 300 µL of TCA were added into the microtube, and the mixture was vortexed and incubated in an oven with a set temperature of 96 ºC for 25 min. After this, the microtubes were placed on ice for 5 min to stop the reaction. Samples were duplicated, so 600 µL was used for every sample and blank, 300 µL in each microplate well. LPO levels are expressed in nmol of MDA to g of FW, at an absorbance of 532 nm, with an extinction coefficient of 1.56 x 105 M^{-1} cm^{-1}, following the Lambert–Beer Law method.

References

73. Aebi, H. (1984). Catalase *in vitro*. *Oxygen Radicals in Biological Systems*, *105*, 121-126. https://doi.org/10.1016/s0076-6879(84)05016-3.

74. Bradford, M.M. (1976). A rapid and sensitive method for the quantitation of microgram quantities of protein utilizing the principle of protein-dye binding. Anal. Biochem. 72, 248–254. https://doi.org/10.1016/0003-2697(76)90527-3

75. Buege, J. A., & Aust, S. D. (1978). Microsomal lipid peroxidation. *Methods in Enzymology*, *52*, 302-310. https://doi.org/10.1016/S0076-6879(78)52032-6.

76. Carlberg, I., & Mannervik, B. (1985). Glutathione reductase. *Methods in Enzymology*, *113*, 484 - 490. https://doi.org/10.1016/s0076-6879(85)13062-4.

77. Ellman, G. L., Courtney, K. D., Andres, V., & Featherstone, R. M. (1961). A new and rapid colorimetric determination of acetylcholinesterase activity. *Biochemical Pharmacology*, *7*, 88–95. https://doi.org/10.1016/0006-2952(61)90145-9.

78. Habig, W. H., Pabst, M. J., & Jakoby, W. B. (1974). *Glutathione S-Transferases. Journal of Biological Chemistry*, *249*(22), 7130-7139. https://doi.org/10.1016/s0021-9258(19)42083-8.

79. Hosokawa M, Satoh T. Measurement of Carboxylesterase (CES) Activities. *Current Protocols in Toxicology* 2002;10:4.7.1-4.7.14.; https://currentprotocols.onlinelibrary.wiley.com/doi/full/10.1002/0471140856.tx0407s10

80 .Gunzler, W. A., & Flohé, L. (1985). Glutathione peroxide. In R. A. Greenland (Ed.), *Handbook of Methods for Oxyradical Research*, (pp. 285-290). https://doi.org/10.1201/9781351072922.
